# Choices Behind Numbers: a Review of the Major Air Pollution Health Impact Assessments in Europe

**DOI:** 10.1007/s40572-018-0175-2

**Published:** 2018-02-05

**Authors:** E. Malmqvist, A. Oudin, M. Pascal, S. Medina

**Affiliations:** 1 Occupational and Environmental Medicine, Lund University, Lund, Sweden; 2 Occupational and Environmental Medicine, Umeå University, Umea, Sweden; 3 Institut de Veille Sanitaire, Saint Maurice, France

**Keywords:** Health impact assessment, Air pollution, HIA, Exposure-response function, Health effects

## Abstract

**Purpose of Review:**

The aim of this review is to identify the key contextual and methodological differences in health impact assessments (HIA) of ambient air pollution performed for Europe. We limited our review to multi-country reviews. An additional aim is to quantify some of these differences by applying them in a HIA template in three European cities.

**Recent Findings:**

Several HIAs of ambient air pollution have been performed for Europe, and their key results have been largely disseminated. Different studies have, however, come up with substantial differences in attributed health effects. It is of importance to review the background contributing to these differences and to quantify their importance for decision makers who will use them.

**Summary:**

We identified several methodological differences that could explain the discrepancy behind the number of attributable deaths or years of life lost. The main differences are due to the exposure-response functions chosen, the ways of assessing air pollution levels, the air pollution scenarios and the study population. In the quantification part, we found that using risk estimates from the European Study of Cohorts for Air Pollution Effects (ESCAPE) instead of the American Cancer Society (ACS) study could nearly double the attributable burden of ambient air pollution.

This study provides some insights into the differential results in previously published HIAs on air pollution in Europe. These results are important for stakeholders in order to make informed decisions.

**Electronic supplementary material:**

The online version of this article (10.1007/s40572-018-0175-2) contains supplementary material, which is available to authorized users.

## Background

During the last decades, epidemiological and toxicological studies have provided enough evidence to conduct health impact assessment (HIA) of air pollution [1, 2]. According to the World Health Organization (WHO), ‘HIA is a practical approach used to judge the potential health effects of a policy, programme or project on a population, particularly on vulnerable or disadvantaged groups’ [3].

HIA estimates the mortality and morbidity rates due to current levels of air pollution and the public health impact that would be expected if the levels of air pollution would change to a certain extent. An HIA thus can estimate the human health impacts of current policy or implemented actions and can thereby become a useful tool for policymakers and planners. Whilst epidemiologists often study the risk of a disease in the presence of exposure relative to risk of a disease in the absence of exposure, a health impact assessor on the other hand often asks the following: how many excess cases of disease will occur in a population of a certain size due to exposure at a certain dose level? [4]. HIAs generally apply exposure-response functions based on risk estimates from the epidemiology literature in order to relate exposure estimated by air pollution assessment and the scenarios for air quality changes (related to the aim of HIA) to a population at risk and its baseline health status (prevalence and incidence of disease) [5].

The first HIA of air pollution in Europe was conducted in Switzerland and shortly followed by a HIA conducted for France, Switzerland and Austria, the Tri-national study [6]. The objective of the Tri-national study was to highlight the hidden costs of traffic by putting a monetary value on the pollution of air. Several HIAs have since been performed for Europe, and their key results have been largely disseminated. HIAs have most commonly been used in large projects such as the Global Burden of Disease by World Health Organization (WHO) [7–9]. The aim of the HIAs conducted up until present has differed resulting in conflicting conclusions in terms of attributed health effects to air pollution. Within the context of its goals, *air pollution scenarios* are created, i.e. current levels compared to air quality guideline levels or current levels compared to increased/decreased levels expected to occur due to certain policies. Some of the underlying HIA aims may be so fundamentally different that they do not allow for comparison of the results attained, i.e. comparing the impact of a specific local road policy in Antwerp, Belgium [10] with a broader climate change policy in five cities in Europe [11].

But even studies with comparable aims have arrived at substantially different attributed health effects. As an example, the Global Burden of Disease (GBD) 2010 reports a worldwide air pollution related death toll that is four times bigger than that of GBD 2000. The differences are according to Organization for Economic Co-operation and Development (OECD) partly explained by increasing air pollution levels in Southeast Asia and diminishing competing risks from poverty related disease and mortality and partly by the methodology applied. Two other methodological reasons for this discrepancy reported by OECD are firstly improved exposure assessments by new remote sensing satellite-based technology instead of spatially scarce numbers of monitoring stations and secondly a more comprehensive and rigorous methodology to assemble and analyse epidemiological findings [12]. Other methodological differences mentioned in the literature are related to the study area [13]. Another difference is the way they expressed the mortality or morbidity impacts such as attributable deaths or life expectancy [13].

## Aim

The aim of this project is to identify key contextual and methodological differences used by the major air pollution related HIAs in Europe in the period of 2000–2012. An additional aim was to quantify some of these differences using a template provided by the project Improving knowledge and Communication for Decision Making on Air Pollution and Health in Europe (APHEKOM).

## Method

The HIAs we reviewed estimate mortality and morbidity rates due to current levels of air pollution in Europe and the public health benefits that would be incurred if the levels of air pollution would decrease to a varying and specified extent.

In this review, we will focus on (a) the exposure-response functions selected (based on risk estimates from the epidemiological literature), (b) air pollution exposure assessment, (c) scenarios of air pollution changes and (d) the selection of population at risk as proposed by a previous review [13]. With *air pollution scenarios*, we mean the hypothetical changes in air pollution levels relating to the aim of the HIAs, i.e. the difference in effect between baseline air pollution levels (often current levels) and those that would (sometimes hypothetical) occur by adhering to a specific policy.

In this article, (1) we present and review the differences in recent HIAs in Europe, and (2) we use a simple exercise using those differences we identified to quantify their importance.

### Part 1: Literature Review

This review is not a systematic review of all HIAs on air pollution. Criteria are large HIAs covering a large proportion of Europe (defined as more than one country) with the aim of guiding international air pollution policies. The HIAs included here have been chosen a priori by the APHEKOM group. In addition, we have added the Health risks of air pollution in Europe (HRAPIE) project and the Tri-national study to fulfil above-mentioned criteria. We have included three WHO-funded GBD studies to identify the methodological changes that have occurred over the years. The information presented in this review is based on available technical reports and articles.

We included the following HIAs:

1. Improving knowledge and Communication for Decision Making on Air Pollution and Health in Europe (APHEKOM)
2. Air Pollution and Health: a European Information System (APHEIS)
3. Clean Air For Europe (CAFE)
4. European Perspectives on Environmental Burden of Disease (hereafter referred to as EBD)
5. Global Burden of Disease due to outdoor air pollution for 2000 (hereafter referred to as GBD2000)
6. Global Burden of disease from urban outdoor air pollution for 2008 (hereafter referred to as GBD2008)
7. Global Burden of disease from ambient air pollution for 2012 (hereafter referred to as GBD2012)
8. Tri-national study
9. HRAPIE

### Part 2: Quantification of Differences

To quantify the reviewed differences, we have used an HIA online tool developed by the APHEKOM project and freely available at http://si.easp.es/aphekom/ to conduct HIAs under alternate assumptions. This online tool provides guidelines and Excel spreadsheets and uses the general framework recommended by WHO for performing an HIA. In this quantification exercise, we have based our analyses on measured annual means of particulate matter with an aerodynamic diameter smaller than 2.5 μm (PM_2.5_) in Stockholm, Budapest and Paris for the years 2004–2006. Thus, this should not be interpreted as a complete HIA but rather as an exercise to quantify and show how methodological differences affect the estimates. The cities were chosen based on their representativeness of European air pollution levels with one city Stockholm, Sweden with relatively low levels (mean levels of PM_2.5_ for 2004–2006 is 9 μg/m^3^); Paris, France with higher levels (mean levels of PM_2.5_ for 2004–2006 is 16 μg/m^3^); and Budapest, Hungary with some of the highest levels of air pollutants (mean levels of PM_2.5_ for 2004–2006 is 34 μg/m^3^) (more details on levels in Table S1 in the Supplement). Budapest was also chosen to help understand how missing PM_2.5_ measurements may influence results when one uses particulate matter with an aerodynamic diameter smaller than 10 μm (PM_10_) measurements as an approximation for the missing values of PM_2.5_ with differing PM_10_/PM_2.5_ ratios.

The three cities have also different age distributions and thus baseline mortality and morbidity rates (see Table 1). As an example, the population in Stockholm was older compared with the other two cities.

**Table 1:** Age, population and mortality distribution in the three cities example for the years 2004–2006

Age (years)	Total population Stockholm (age distribution % of population)	Total mortality Stockholm 2004–2006	Total population Paris (age distribution % of population)	Total mortality Paris 2004–2006	Total population Budapest (age distribution % of population)	Total mortality Budapest 2004–2006
30–34	114,331 (14.3)	183 (0.6)	556,968 (14.3)	1094 (0.9)	147,248 (12.7)	309 (0.5)
35–39	102,236 (12.8)	212 (0.6)	504,035 (12.9)	1526 (1.3)	124,847 (10.8)	456 (0.7)
40–44	93,698 (11.7)	289 (0.9)	470,741 (12.1)	2332 (2.0)	95,126 (8.2)	758 (1.1)
45–49	83,059 (10.3)	451 (1.4)	428,835 (11.0	3471 (2.9)	104,103 (9.0)	1634 (2.4)
50–54	77,261 (9.6)	776 (2.3)	410,073 (10.5)	5253 (4.5)	145,540 (12.6)	3546 (5.2)
55–59	81,444 (10.2)	1369 (4.3)	404,174 (10.4)	6886 (5.9)	125,165 (10.8)	4254 (6.2)
60–64	66,272 (8.3)	1656 (5.0)	274,860 (7.1)	6320 (5.4)	112,722 (9.7)	5237 (7.6)
65–69	45,195 (5.6)	1876 (5.7)	215,004 (5.5)	7459 (6.4)	83,370 (7.2)	5504 (8.0)
70–74	37,678 (4.7)	2476 (7.5)	200,488 (5.2)	10,565 (9.1)	73,578 (6.4)	7459 (10.9)
75–79	36,131 (4.5)	3937 (11.9)	178,049 (4.6)	14,594 (12.5)	66,662 (5.8)	10,623 (15.5)
80–84	33,482 (4.2)	6362 (19.2)	138,960 (3.6)	19,248 (16.5)	47,910 (4.1)	12,552 (18.3)
> 85	29,585 (3.7)	13,558 (40.9)	108,313 (2.8)	37,895 (32.5)	30,317 (2.6)	16,281 (23.7)
Total	800,372	33,145	3,890,498	163,888	1,156,588	68,613

We made the following methodological choices for our alternate HIA calculations:

- We derived the exposure response-function from all-cause mortality risk estimates in the American Cancer Society Study by Pope et al. (hereafter referred to as ACS study) that reported hazard ratios (HR) of 1.06 (CI 1.02–1.11) per 10 μg/m^3^ increment of PM_2.5_ for total mortality [14]; alternatively, we used the new European Study of Cohorts for Air Pollution Effects (ESCAPE) results by Beelen et al. 2014, hereafter referred to as ESCAPE study with an HR of 1.07 (CI 1.02–1.13) per 5 μg/m^3^ increment of PM_2.5_ [15•] for natural all-cause mortality after transforming the ESCAPE HR to a 10-μg/m^3^ increment of PM_2.5_ (HR of 1.14 (95% CI 1.09–1.20).
- Levels of air pollutants can depend not only on the accuracy of modelling (which we unfortunately could not access) but also on chosen strategies with missing measurement data. Some parts of the world lack/lacked PM_2.5_ measurements to deal with this issue; some of the HIAs we reviewed (see Table S4 in the Supplement) converted the measured pollutant into the desired pollutant by a simplified ratio. This ratio varied by HIA (see Table S4 in the Supplement). We evaluated the changes in estimates when using different conversion rates from PM_10_ to PM_2.5_, specifically using 0.5 or 0.7, to replace missing data for PM_2.5_ based on PM_10_ measures.
- The attributed health effects of air pollution will hypothetically depend on what size changes are expected, the *air pollution scenarios*. We evaluated three PM_2.5_ scenarios found in our literature review: levels corresponding to WHO air quality guidelines (10 μg/m^3^) (scenario 1 in our literature review), a general 5 μg/m^3^ reduction from current levels (scenario 3 in our literature review) or setting the expected air pollution at zero (scenario 6 in our literature review).
- We briefly evaluated the role the composition of the study population plays in terms of age distribution and baseline mortality by studying three different European cities, Stockholm, Paris and Budapest.

## Results

### Part 1: Literature Review

#### Exposure-Response Functions Based on Risk Estimates From Epidemiology

Most studies used risk estimates for cause-specific mortality for particulate matter (PM) as exposure-response function. There were, however, some differences in the risk estimates chosen as well as the underlying causes (diseases) behind mortality. APHEIS, CAFE, EBD and GBD 2000 and 2008 used risk estimates from Pope et al. [14] and only modelled HIAs for cardiopulmonary and lung cancer mortality. APHEKOM used a later study by Pope et al. [16] on cardiovascular mortality and GBD 2012 used Burnett et al. for integrated exposure-response function (based on risk estimates of mortality from ischemic heart disease, cerebrovascular disease (stroke), chronic obstructive pulmonary disease (COPD) and lung cancer in adults and acute lower respiratory infection in children) [17•].

Other studies used risk estimates for all-cause mortality or a combination of all cause and cause-specific mortality (see Table S2 in the Supplement). Whilst all-cause mortality should include all causes of death, this is sometimes limited to natural all-cause mortality excluding deaths by accidents or trauma. Morbidity due to long-term exposure to PM was only included in some but not all studies (see Table S3 in the Supplement).

The above-mentioned morbidity/mortality rates are for long-term exposure (months to years) to PM, but air pollution may also exert its effects also after short-term exposures (days). The most commonly used short-term effect of PM in HIA is infant mortality (for more, see Table S3 in the Supplement). Both CAFE and GBD (all) addressed infant mortality. In CAFE, they relied on Woodruff et al. with a relative risks (RR) of 1.04 for mortality in the first 2 months of life with each 10 μg/m^3^ increase in PM_10_ [18]. In GBD2000, 2008 and 2012, they used a meta-analysis combining five times series that estimated a meta-RR for respiratory mortality in children under 5 years of age [1.017; 95% confidence interval (CI) 1.003, 1.030] [19]. In GBD 2012, the respiratory mortality for children under 5 years of age is included in the integrated risk function from Burnett et al. [17•]. In addition, some HIAs included short-term effects on chronic bronchitis and hospitalizations for respiratory/cardiac diseases (see Table S3 in the Supplement). Health effects due to pollutants other than PM (PM_10_ and PM_2.5_) were only considered for ozone in APHEKOM, CAFE, EBD and HRAPIE and for NO_2_ in HRAPIE [1].

#### Air Pollution Assessment

The levels of air pollutants were generated either from measurements from monitoring stations or by modelling (see Table S4 in the Supplement). In the APHEIS and APHEKOM and GBD 2000 and 2008 projects, PM_2.5_ was assessed from monitoring stations and if PM_2.5_ was not measured, several studies used PM_10_ measurement data and applied varying conversion rates (0.5–0.7) [7, 8, 20–22]. The Tri-national study used a combination of measurements of total suspended particulates, black smoke, PM_10_ and nitrogen oxides (NO_*x*_) (for all countries) and a dispersion model (only available for Switzerland) to assess exposure to PM_10_. In HRAPIE, the experts concluded that Europe-wide modelling for particles is only available for PM_2.5_; so, in cases where health effects are expressed against PM_10_ in the literature, a conversion has to be employed to assess the equivalent impact per unit of PM_2.5_. This is achieved by multiplying the exposure-response function for PM_10_ by a factor of 1.54, assuming a PM_2.5_/PM_10_ ratio of 0.65 [1].

For CAFE, PM_2.5_ was modelled using the European Monitoring and Evaluation Programme (EMEP) Eulerian model with a resolution of 50 × 50 km including only anthropogenic emissions [23–26]. For EBD, the pollutants were modelled by the European Topic Centre on Air and Climate Change (ETC/ACC) with 10 km × 10 km resolution [19]. For GBD 2012, annual mean estimates of PM_2.5_ were modelled with a grid of 0.1° × 0.1° (approximately 11 × 7 km for a centroid in Europe) [9, 21, 27].

Ozone assessment was only included in the APHEKOM, CAFE, HRAPIE and EBD projects. In APHEKOM, ozone was assessed by times series from monitoring stations, using the daily maximum of the 8 h mean. For short-term effects, measurements for a whole year were used and for long-term effects, measurements were restricted to data from April to September [28]. In CAFE, ozone above a cut-off point of a daily maximum 8-h mean of 35 ppb (SOMO35) was calculated using the EMEP Eulerian model with a resolution of 50 × 50 km including reported anthropogenic emissions [23–25, 29]. For EBD, the ozone levels (SOMO35) were modelled using AirBase data and air quality maps [19]. Also, the HRAPIE experts recommended an ozone cut-off concentration of 35 ppb.

Levels of pollutants differ by included areas and years. Emissions of air pollutants in the EU have generally dropped from 2002 to 2011 by 14% for PM_10_ and by 16% for PM_2.5_ mainly due to tighter vehicle emission standards; however, increasing urbanization makes more people exposed to higher levels [12].

#### Air Pollution Scenarios

The HIAs relate the health impact of the assessed air pollution levels during the study period to a counterfactual level that is assumed by an *air pollution scenario*. In APHEKOM, APHEIS and GBD2008, the *air pollution scenario* (scenario 1) assumes that air pollution health effects could have been avoided if the mean annual WHO Air Quality Guidelines (AQG) values of PM_2.5_ = 10 μg/m^3^ had been implemented (used as counterfactual values) [8, 19, 20, 30]. The Tri-national study used a counterfactual level of 7.5 μg/m^3^ for PM_10_ (scenario 2)_._ APHEIS and APHEKOM also studied other reduction scenarios for PM or ozone such as a reductions of 5 μg/m^3^ modeled/measured levels at study year [20] (scenario 3). For CAFE, the RAINS model was used with two different scenarios the first, scenario 4, is called the current legislation (CLE) which assumes the successful implementation of all presently decided emission-related legislation in all countries of the EU-25 [31]. The second scenario, scenario 5, is the maximum technical feasible reduction case (MFR) [31] which also assumes full implementation of the presently available most advanced technical emission control measures in the year 2020 (but specific reduction levels of scenarios 4 and 5 are varying in time and no specific reduction levels can be presented here) [23–26]. For EBD, no threshold was used [19] and levels were compared to a scenario in which air pollution levels would be reduced down to zero, scenario 6. For GBD 2000, the burden of disease was estimated with respect to counterfactual concentrations of 7.5 μg/m^3^ for PM_2.5_ or 15 μg/m^3^ for PM_10_, scenario 7_._ In the GBD 2012, the counterfactual concentration, scenario 8, was selected to be between 5.8 and 8.8 μg/m^3^, depending on area [17•]. HRAPIE experts recommend assuming benefits of any reduction in exposure, including at very low modelled levels of PM2.5 [1] but no specific scenarios are presented.

#### Study Populations

The APHEKOM and APHEIS projects were based on 25 and 26 cities, respectively, in 12 countries and only included members of the population over 30 years of age [28]. For CAFE, the study area was based on the EU-25 countries [23, 25, 31]. The EBD was based on six countries: Belgium, Finland, France, Germany, Italy and the Netherlands [19, 30]. The GBDs were made globally but results are also presented regionally, European data were divided into (1) high-income and (2) low- and middle-income countries. For the GBD 2000 and 2008, only urban air pollution was accounted for by restricting to cities with more than 100,000 inhabitants and to national capitals. This was extended to whole populations (regardless of the number of inhabitants in an area) in GBD 2012 [7, 8, 32]. The study populations are further described in the Supplement. HRAPIE experts recommended estimation of most mortality effects in adult populations (age 30+ years) as most of the health effect evidence comes from studies that focus on populations in this age group [1]. The Tri-national study also used this cut-off for their mortality studies [6].

#### Presentation of Reviewed Results

More detailed definitions are provided in the Supplement. In APHEKOM, APHEIS, GBD 2000, GBD 2008 and GBD 2012, the results are presented as *premature attributable deaths* [20]. In GBD 2000, some results were also presented as DALY (consisting only of *years of life lost* (YLL) due to premature mortality) [32]. In CAFE, the mortality effects were expressed as change in longevity by *years of life lost* and in the cost-benefit analysis *value of a life year.* But due to the methodological difficulties of assessing different age-specific *values of a life year*, attributable deaths were also calculated. Due to methodological problems, life tables were not used in CAFE [23–25]. In EBD, results were presented as DALYs, without age weighting or discounting [19]. No time lags were used between assumed changes in pollution and consequent changes in death rates in any of the studies included in the present review (Table 2).

**Table 2:** Definitions and results presented by the HIA authors

Study	Results	Definition of impact
AHEKOM	19,000	Deaths^a^
APHEIS	11,375	Deaths^a^
CAFE (CBA)	8.1	YLL^b^ (months)
EBD	4500–10,000	DALYs^c^/million people
GBD 2005^d^	2 3000	Deaths^a^
GBD 2008^d^	67,000	Deaths^a^
GBD 2012^d^	279,000	Deaths^a^
HRAPIE	n.a.	
Tri-national	40,000	Deaths^a^

*n.a.* not applicable

^a^Deaths refer to premature attributable deaths here due to mainly long-term air pollution exposure

^b^Years of life lost

^c^Disability adjusted life years

^d^In the GBD reports, we used the EUR A countries

### Part 2: Quantification

#### Exposure-Response Functions Based on Risk Estimates From Epidemiology

We illustrated the implication of using different risk estimates as exposure-response functions by comparing risk estimates based on the ACS study with those based on the ESCAPE study. Using the ESCAPE risk estimates and different scenarios changed the results dramatically (Table 3). Using the risk estimate from ESCAPE more than doubled the estimated attributed deaths (see Table 3 below and Figs. S1, S2 and S3 in the Supplement for more detailed results).

**Table 3:** Total number of attributed death and population weighted (per 100,000) for the three cities for three air pollution (PM_2.5_) scenarios and two different exposure-response functions

Air pollution scenarios in literature review	E-R function from ESCAPE^1^ # of deaths (deaths per 100,000)	E-R function from ACS^2^ # of deaths (deaths per 100,000)
	Total	Total
Scenario 1; WHO AQG (10 μg/m^3^ PM_2.5_)	9234 (608)	4375 (292)
Scenario 3; 5 μg/m^3^ reduction of PM_2.5_	4617 (276)	2090 (126)
Scenario 6; null (0 μg/m^3^) PM_2.5_	16,965 (1059)	8211 (518)
	Budapest	Budapest
Scenario 1; WHO AQG (10 μg/m^3^ PM_2.5_)	6108 (528)	2952(255)
Scenario 3; 5 μg/m^3^ reduction of PM2.5	1450(125)	657 (57)
Scenario 6; null (0 μg/m^3^) PM_2.5_	8167(706)	4079 (353)
	Paris	Paris
Scenario 1; WHO AQG (10 μg/m^3^ PM_2.5_)	3126(80)	1423(37)
Scenario 3; 5 μg/m^3^ reduction of PM2.5	2466(63)	1116(29)
Scenario 6; null (0 μg/m^3^) PM_2.5_	7517 (193)	3543 (91)
	Stockholm	Stockholm
Scenario 1; WHO AQG (10 μg/m^3^ PM_2.5_)	0 (0)	0 (0)
Scenario 3; 5 μg/m^3^ reduction of PM2.5	701(88)	317(40)
Scenario 6; null (0 μg/m^3^) PM_2.5_	481(160)	589 (74)
1. HR of 1.14 per 10 μg/m^3^ increase in PM_2.5_ [15] 2. HR of 1.06 per 10 μg/m^3^ increase in PM_2.5_ [14]		

#### Air Pollution Assessment

We evaluated the consequences of using different conversion rates for PM_10_ to PM_2.5_ by using the ratios (0.5 or 0.7) to estimate PM_2.5_ from PM_10_. Only Budapest lacked PM_2.5_ measurements; using a ratio of 0.5 instead of 0.7 did not change the results in scenario 3 (as expected since the ratio does not affect an absolute reduction) but reduced the attributed death to 156 (per 100,000) instead of 255 (per 100,000) in scenario 1 and 259 instead of 353 in scenario 6 (numbering of scenarios can be found below).

#### Air Pollution Scenarios

We illustrate the effects using three scenarios:

- The compliance with WHO Air Quality Guidelines (10 μg/m^3^) (scenario 1) used in GDB 2008, APHEIS and APHEKOM, which is the most conservative approach
- The 5 μg/m^3^ reduction used in APHEIS and APHEKOM (scenario 3), which could somewhat be representable for a hypothetical general reduction in emissions (with new emission standards or low emission zones)
- The null air pollution scenario from EBD (scenario 6) (and in one calculation in CAFE), which represents the least conservative approach

#### Study Populations

We conducted stratified analyses for Stockholm, Paris and Budapest, and the study population has a large impact due to the combination of exposure levels and demography as illustrated in Figs. S1, S2 and S3 in the Supplement.

## Discussion

Our review has identified large differences in HIA methods applied across studies. Regarding the outcomes, some studies have focused on mortality; others have also included effects on morbidity such as hospitalization for respiratory diseases. The HIAs also differ with respect to long-term or short-term health effects or both. As epidemiological evidence is evolving, the options of what to include in an HIA increase and will thus influence the results that an HIA produces. The results of the HIA will also heavily depend on which epidemiological mortality study/studies were selected to derive the exposure response function. In our calculations, we quantified some of these differences by using more recently published results by ESCAPE on mortality to replace the commonly used mortality effect estimates from the ACS study (see Figs. S1, S2 and S3 in the Supplement). We illustrated that, when using the ESCAPE risk estimates, the estimated number of deaths attributable to air pollution nearly doubled compared with using the risk estimates based on ACS study [14]. Whilst the ACS study [14] studied total mortality, the ESCAPE study [15•] limited death to natural cause mortality, hence excluding deaths by accidents or crimes. Furthermore, whilst the ACS study relies solely on exposure differentials between cities, the ESCAPE study used an exposure assessment that also included within city variations in exposure. Jerrett et al. [33] revised the data from ACS study with a model of more spatially refined exposures and reported that effect estimates increased to 1.14, 95% CI: 1.11–1.17 per 10 μg/m^3^ increment in PM_2.5_. Interestingly, these estimates are much more in line with the estimates derived by ESCAPE emphasizing the importance of the choice of exposure assessment models when deriving exposure response functions. There was some difficulty in reviewing morbidity health outcomes and whether they were related to long- or short-term (acute) effects in the HIAs due to lack of reporting. Sometimes, estimates from a short-term effect were used as a proxy for long-term effects, such as for ‘days of restricted activity’, but this was not always clearly stated.

Attributable death in epidemiology refers the number of (premature) deaths attributable to the factor(s) of interest. But this does not mean that removal of exposure will permanently reduce the number of death. Death are due to happen anyway but can be postponed, and exposure to air pollution is but one of several risk factors that can contribute to development of chronic diseases and ultimately death [34]. Air pollution is a mixture of gases and particles in the air, and each component can have different health effects. Primarily, previous studies have focused on the impact of particle exposure measured as PM_2.5_ or PM_10_; however, some studies have also included the effects of ozone. NO_*x*_ or nitrogen dioxide (NO_2_) is not accounted for, in order to avoid double counting mixture effects, as PM and NO_*x*_/NO_2_ may have similar or same sources and/or are markers for same mixture effect of air pollution. However, increasing evidence of independent effect of NO_2_, HRAPIE opened up for using NO_2_ estimates but to avoid double counting [1].

Moreover, modelled versus measured air pollutant levels both have advantages and disadvantages. Modelled levels depend on many factors used as input into the model, for example only including anthropogenic emissions such as in CAFE will results in lower total levels than when accounting for both anthropogenic emissions and biogenic emissions such as in EBD and GBD 2012. This might impact the counterfactual level if using a cut-off such as adhering to WHO air quality guidelines. The accuracy of the modelled levels will also depend on the spatial (or temporal) resolution of the model [33•, 35]; CAFE used a resolution of 50 × 50 km whilst EDB and GDB 2012 used 10 × 10 km and 0.1° × 0.1° (which is approx. 7 × 11 km in Europe). Measured levels on the other hand have an even cruder spatial resolution often depending on one or few measurements per city for the whole city population, which might reflect poorly exposure at a residential address often used nowadays in epidemiological studies [33•, 35]. Moreover, PM_10_ was transformed to PM_2.5_ by using a ratio in several studies, an approach with evident weaknesses. The PM_10_/PM_2.5_ ratio varies by study. Furthermore, some studies included effects of ozone whilst other HIAs were restricted to particles only. The negative health impact estimated in the HIA would increase if pollutants other than PM and ozone were included but a conservative approach is often used to avoid double counting of effects of shared sources of exposure that will produce an overestimation of the health impacts [6].

Study year could also play a role; PM and ozone levels vary between years not only due to policy actions but also due to meteorological factors. Careful consideration should be made especially for years with abnormal mean levels of air pollutants. This can be accounted for by using means over several years.

As discussed previously by Medina et al., there are two different ways of assessing the quantification of health impact in the HIAs: the predictive and the counterfactual approaches. The predictive approach assesses future health consequences of public policies applied today or in a given time frame; it requires making assumptions about future trends in demographics and health status, time needed to achieve pollutant reduction and the time lag between pollutant reduction and improved health. The counterfactual approach compares the current health status of the population to a hypothetical one had the same population not been exposed, and it uses the epidemiological concept of an attributable fraction to generate its estimates. Whereas the counterfactual approach might be more useful to quantify the health burden of air pollution and encourage action, the predictive approach might be more useful to show stakeholders the benefits of implementing a specific concrete policy they developed [36]. The predictive approach will however rely on additional assumptions, such as the success of policy implementations or baseline disease rate changes due to other causes, and it is important that those are stated clearly.

Furthermore, populations may differ in terms of baseline risk factors including age resulting in different baseline morbidity/mortality rates for each country. It is a well-known fact that effect modifiers limit the generalizability of epidemiologic results across different populations [37]. Source of baseline health data, disease definitions and study period modeled can strongly influence results and the importance of the latter has been highlighted in several reviews [38, 39]. Generally, this is less of a problem in Europe where consistent definitions of and procedures to collect disease/mortality data are available, but ICD code changes over time can still influence results. Other countries or health end points might have less standardized data available. Generally, age-specific mortality rates by country are widely available and in Europe, this is also true for morbidity rates.

When considering and aggregating number of deaths, it should be kept in mind that, eventually, everyone dies but those at older ages are at higher risks [23–25]. This has been exemplified in our quantification, and whilst it is easily understood that attributable deaths are larger for Budapest with a larger annual mean for PM_2.5_, it might be harder to understand for Stockholm with a lower annual mean for PM_2.5_ than Paris. This can be explained by the differences in age distributions, i.e. Stockholm has an older population than Paris and thus a larger population vulnerable and at risk of dying when exposed. This might also be true for unmeasured risk factor distributions that differ between cities. This issue depends on how we present our results, i.e. as number of premature deaths or disability adjusted life years (DALY). For more detailed discussion of this issue and recommendation for the use of terms such as ‘average loss of life expectancy’, refer to [34].

There is an ongoing discussion whether future years of healthy life are valued less than those at present time (discounting) and if a year lived as a young adult is worth more than a year lived at younger or older ages. This can be accounted for by discounting or age weighting the results and is optional when calculating DALYs. For example the EBD has chosen not to discount or age weight their estimates as it often makes health impacts in children weigh less with the argument that this would not be in line with the priorities set in the European Health Action Impact plan (WHO 2010b) that made children’s health a priority [19]. Given the European Health Action Plan, it can also be argued that more negative outcomes by air pollution in early years, i.e. birth outcomes, could be included in HIAs of air pollution.

Finally, we would like to make some suggestions for future HIAs. It is important to carefully consider the exposure assessment methods and use better and/or cheaper models validated with measurement data at several sites. The effect of using a ratio to transform PM_10_ to PM_2.5_ will be less of a problem in the future when PM_2.5_ measurements are more widespread. It is also important to note that the field of air pollution epidemiology is constantly evolving with evidence for new health outcomes (i.e. reproductive outcomes, autism, Alzheimer, diabetes, cognitive functions in children), and new effect estimates are derived and can be used for updating exposure response functions for HIAs such as in ESCAPE [15•]. We also need to consider in which situations one of the two HIA approaches may be most relevant: predictive or counterfactual approach. The aim of HIAs might also be different and should be considered when interpreting results.

## Conclusion

We have identified several methodological differences that explain the discrepancy of the estimated number of attributable deaths or DALYs in recent HIAs. The main differences we identified are the exposure response functions used, the ways air pollution levels were assessed, the aim of the HIA (and thus the air pollution scenarios chosen) and the risk factor characteristics of the study population. By using the dose-response function from the ESCAPE project [15•], the estimated number of deaths attributable to air pollution nearly doubled compared with the dose-response function from the ACS study [14]. Making assumptions that go into these results more transparent and showing different scenarios is important for stakeholders in order to take informed decisions. Thus, both aims and assumptions need to be clearly described when presenting results from HIA and it would be even better to provide easy data and model access to anyone who wishes to change these assumptions.

## Electronic Supplementary Material

ESM 1 (DOCX 72 kb)

## Abbreviations

ACS: American Cancer Society
AHSMOG: Loma Linda University Adventist Health and Smog study
APED: Air pollution epidemiology database
APHEA: Air Pollution and Health: a European Approach project
APHEKOM: Improving knowledge and Communication for Decision Making on Air Pollution and Health in Europe
APHEIS: Air Pollution and Health: a European Information System
APHENA: Air Pollution and Health: a European and North American approach study
CAFE: Clean Air for Europe
CI: Confidence interval
DALY: Disability-adjusted life year
ESCAPE: European Study of Cohorts for Air Pollution Effects
EBD: European Perspectives on Environmental Burden of Disease
EU: European Union
GBD2000: The Global Burden of Disease due to outdoor air pollution for 2000
GBD2008: Global Burden of Disease from urban outdoor air pollution for 2008
GBD2012: Global Burden of Disease from ambient air pollution for 2012
GBD 2010: Global Burden of Disease 2010 study
HR: Hazard ratio
HIA: Health impact assessment
HRAPIE: Health risks of air pollution in Europe
ICD: International Classification of Diseases
IIASA: International Institute for Applied Systems Analysis
MRAD: Minor restricted activity day
NO2: Nitrogen dioxide
NO*x*: Nitrogen oxides
O3: Ozone
OECD: Organization for Economic Co-operation and Development
PM: Particulate matter
PM10: Particulate matter with an aerodynamic diameter smaller than 10 μm
PM2.5: Particulate matter with an aerodynamic diameter smaller than 2.5 μm
ppb: Parts per billion
RAD: Restricted activity day
REVIHAAP: Review of evidence on health aspects of air pollution project
RR: Relative risk
SD: Standard deviation
WHO: World Health Organization
YLL: Years of life lost
