# Supplementary material for: Choices Behind Numbers: a Review of the Major Air Pollution Health Impact Assessments in Europe
Source: Curr Environ Health Rep. 2018 Feb 5;5(1):34–43. doi: 10.1007/s40572-018-0175-2 (PMC5876343; doi:10.1007/s40572-018-0175-2)
Supplement: Supplementary file 1 — (DOCX 72 kb) [file 40572_2018_175_MOESM1_ESM.docx]

# Supplement

**Table S1. Levels of PM_2.5_ (µg/m^3^) during the years 2004-2006 in the three cities examples**

| PM_2.5_ (µg/m^3^) | Budapest (from PM_10_) | Paris | Stockholm |
| --- | --- | --- | --- |
| Minimum (µg/m^3^) | 6 | 4 | 2 |
| Maximum (µg/m^3^) | 131 | 88 | 46 |
| Mean (SD) (µg/m^3^) | 34 (19) | 16 (9) | 9 (6) |
| Valid data (%) | 99 | 100 | 96 |

**Table S2. Effect estimates by study for all-cause mortality and cause-specific mortality and PM**

| HIA  Study year | All-cause mortality | Risk estimate  (95% CI)  per 10 µg/m^3^ PM | Cause-specific mortality  (outcome) | Risk estimate  (95% CI)  per 10 µg/m^3^  PM |
| --- | --- | --- | --- | --- |
| APHEKOM  2004-2006 | Pope et al. 2002 | 1.06  (1.02-1.11) | Pope et al. 2004  (Cardiovascular) | 1.12  (1.08-1.15) |
| APHEIS  2000 | Pope et al. 2002 | 1.06  (1.02-1.11) | Pope et al. 2002  (a) Cardio- pulmonary b) Lung cancer) | a) 1.06  (1.02-1.10)  b) 1.08  (1.01-1.16) |
| CAFE  2000 | Pope et al. 2002 | 1.06  (1.02-1.11) | none | none |
| EDB  2005 | none | none | Pope et al. 2002  (a) Cardio- pulmonary b) Lung cancer) | a) 1.06  (1.02-1.10)  b) 1.08  (1.01-1.16) |
| GBD  2000 | none | none | Pope et al. 2002  (a) Cardio- pulmonary b) Lung cancer) | a) 1.06  (1.02-1.10)  b) 1.08  (1.01-1.16) |
| GBD  2008 | none | none | Pope et al. 2002  (a) Cardio- pulmonary b) Lung cancer) | a) 1.06  (1.02-1.10)  b) 1.08  (1.01-1.16) |
| GBD  2012 | none | none | Burnett et al. 2014 | Integrated risk function on mortality from ischemic heart disease, cerebrovascular disease (stroke), chronic obstructive pulmonary disease (COPD), and lung cancer in adults and acute lower respiratory infection in children |
| HRAPIE | Hoek et al. (2013) | 1.06  (1.04- 1.08) | none | none |
| Tri-National  1996 | Pope et al. 1995 Dockery et al. 1993 | 1.04  (1.03-1.06 | none | none |

**Table S3. Effect estimates by study related to morbidity from long-term PM (PM_2.5_/PM_10_) effects, morbidity, and from ozone**

| HIA  Study year | Morbidity from long-term PM effects | Morbidity from short-term PM effects  (outcome) | Morbidity/mortality from ozone |  |
| --- | --- | --- | --- | --- |

| AHEKOM  2004-2006 | none | Hospitalization for respiratory and cardiac diseases) (Apheis-3 review) | Respiratory hospitalization Andersson et al. 2007  Respiratory mortality  (Jerrett et al. 2009) |
| --- | --- | --- | --- |
| APHEIS  2000 | none | Hospitalization for respiratory and cardiac diseases) (Apheis-3 review)) |  |
| CAFE  2000 | Respiratory diseases and restricted activity days CAFE review | Woodruff et al. 1997  (infant mortality) | Mortality based on US EPA evaluation |
| EDB  2005 | Respiratory diseases and restricted activity days (CAFE review) | Hospitalization for respiratory and cardiac diseases) (Apheis-3 review))  Bronchitis (US AHSMOG study) | Children resp. symptoms (Hurley et al. 2005)  Minor restricted activity days and mortality (WHO, 2006) |
| GBD  2000 | none | Meta-analysis on five time series studies  (infant mortality) | none |
| GBD  2008 | none | Meta-analysis on five time series studies  (infant mortality) | none |
| GBD  2012 | none | Burnett et al. 2014  (infant mortality) | none |
| HRAPIE | none | none | Mortality (APHENA review)  adjusted for PM_10_ |
| Tri-National  1996 | Tri-national review | none | none |

**Table S4. Differences in study populations pollutants, exposure assessment, particle ratios, and scenarios**

| Study | Study population by country, year, and inclusion criteria | Pollutants | Exposure assessment | Ratio PM_2.5_/PM_10_ | Air pollution scenario/s for counterfactual levels of PM_2.5_ or PM_10_ |
| --- | --- | --- | --- | --- | --- |
| AHEKOM | 25 European cities from 12 countries, 2004-2006, >30 years | Ozone  PM_2.5_ | Measured | 0.7 | 1) PM_2.5_=10 µg/m^3^  2) reductions of 5 µg/m^3^ from level at study year |
| APHEIS | 26 European cities from 12 countries, 2000,  >30 years | PM_2.5_ | Measured | 0.7 | 1)PM_2.5_=10 µg/m^3^  2)reductions of 5 µg/m^3^ |
| CAFE | EU-25 countries, 2000, all | Ozone  PM_2.5_ | Modelled | n.a | 1) PM_2.5_ =0 µg/m^3^ or 2) Legislation scenarios |
| EBD | Belgium, Finland, France, Germany, Italy and the Netherlands,  2005, all | Ozone  PM_2.5_ | Modelled | n.a | PM_2.5_=0 µg/m^3^ |
| GBD 2000 | Globally (regionally European by country income) only cities >100 000 and national capitals, 2000, all | PM_10_ | Measured | 0.5 | PM_2.5_ reduced to 7.5 µg/m^3^ or PM_10_ reduced to 15 µg/m^3^ |
| GBD 2008 | Globally with area-specific results only cities >100 000 and national capitals, 2008, all | PM_10_ | Measured | Differ by study area i.e. 0.73  for Europe HIC | PM_2.5_ reduced to10 µg/m^3^ |
| GBD 2012 | Globally with area specific results | PM_2.5_ | Modelled | n.a | PM_2.5_ =5.8- 8.8 μg/m^3^ |
| HRAPIE | Europe | Ozone and PM_2.5_ | Modelled | 0.65 | n.a |
| Tri-National | France, Austria and Switzerland, 1996 | PM_10_ | Measured and modelled | n.a | PM_10_=7.5 μg/m^3^ |


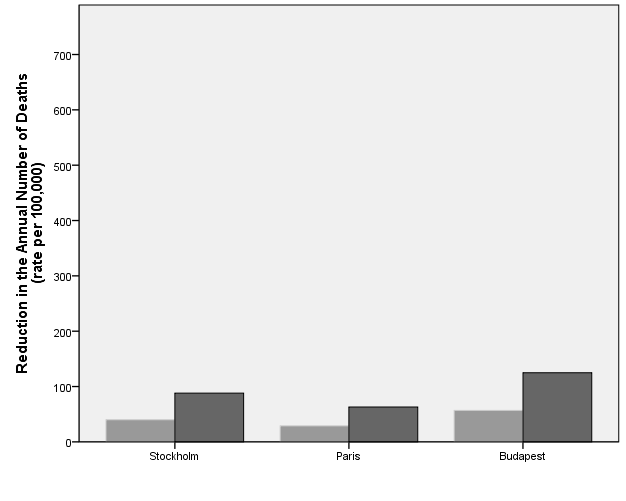


**Figure S1. Air pollution reduction by 5 µgm^-3^ PM_2.5_ results using exposure response function by ACS (1) in *light grey bars* and by ESCAPE (2) in *dark grey bars*.**


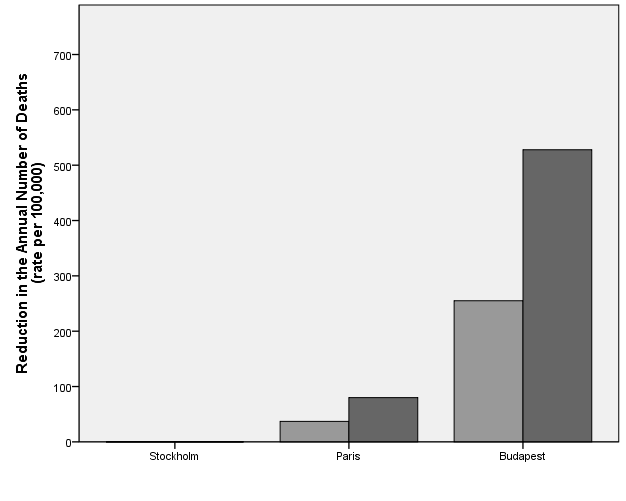


**Figure S2. Reduce PM_2.5_ levels to 10 µgm^-3^. *Light grey bars* represents exposure response functions by ACS (1) and *dark grey bars* represents exposure response functions by ESCAPE (2).**


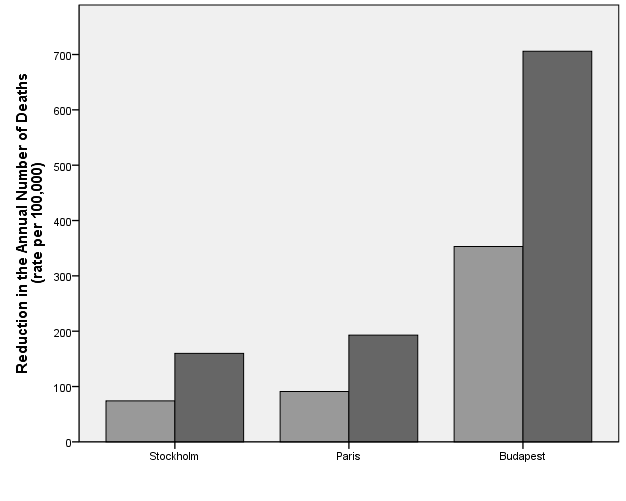


**Figure S3. Reduce PM_2.5_ levels to 0 µgm^-3^. *Light grey bars* represents exposure response functions by ACS (1) and *dark grey bars* represents exposure response functions by ESCAPE (2).**

**Definitions**

DALY: The disability-adjusted life year [DALY] is a measure of number of years lost due to ill-health, disability or early death(3).

YLL: Years of Life Lost [YLL] an estimate of the average years a person would have lived if he or she had not died prematurely(4)

VSL: Value of a statistical life (year) [VSL] estimating the benefits of policies that save lives by giving it a monetary value i.e., by asking people to say how much they would pay to reduce their risk of dying(5).

**References**

1. Pope CA, 3rd, Burnett R, Thun M, Calle E, Krewski D, Ito K, Thurston G. Lung cancer, cardiopulmonary mortality, and long term exposure to fine particulate ai rpollution. . JAMA. 2002(287):1132-41.

2. Beelen R, Raaschou-Nielsen O, Stafoggia M, Andersen ZJ, Weinmayr G, Hoffmann B, et al. Effects of long-term exposure to air pollution on natural-cause mortality: an analysis of 22 European cohorts within the multicentre ESCAPE project. Lancet (London, England). 2014;383(9919):785-95.

3. WHO. DALY definition 2017 [Available from: <http://www.who.int/healthinfo/global_burden_disease/metrics_daly/en/>.

4. Gardner JW, Sanborn JS. Years of potential life lost (YPLL)--what does it measure? Epidemiology (Cambridge, Mass). 1990;1(4):322-9.

5. Alberini A. What is a life worth? Robustness of VSL values from contingent valuation surveys. Risk analysis : an official publication of the Society for Risk Analysis. 2005;25(4):783-800.
